# Measuring test-retest reliability (TRR) of AMSTAR provides moderate to perfect agreement – a contribution to the discussion of the importance of TRR in relation to the psychometric properties of assessment tools

**DOI:** 10.1186/s12874-021-01231-y

**Published:** 2021-03-11

**Authors:** Stefanie Bühn, Peggy Ober, Tim Mathes, Uta Wegewitz, Anja Jacobs, Dawid Pieper

**Affiliations:** 1 Institute for Research in Operative Medicine, Faculty of Health - School of Medicine, Witten/Herdecke University, Ostmerheimer Str. 200, Building 38, 51109 Cologne, Germany; 2 LIFE Child, LIFE Leipzig Research Center for Civilization Diseases, Leipzig University, Ph.-Rosenthal-Str. 27, 04103 Leipzig, Germany; 3 Federal Institute for Occupational Safety and Health (BAuA), Nöldnerstr. 40-42, 10317 Berlin, Germany; 4 Federal Joint Committee (Healthcare), Gutenbergstraße 13, 10587 Berlin, Germany

**Keywords:** Test-retest-reliability, AMSTAR, Systematic reviews, Reliability, Psychometric properties, Quality assessment tool

## Abstract

**Background:**

Systematic Reviews (SRs) can build the groundwork for evidence-based health care decision-making. A sound methodological quality of SRs is crucial. AMSTAR (A Measurement Tool to Assess Systematic Reviews) is a widely used tool developed to assess the methodological quality of SRs of randomized controlled trials (RCTs). Research shows that AMSTAR seems to be valid and reliable in terms of interrater reliability (IRR), but the test retest reliability (TRR) of AMSTAR has never been investigated. In our study we investigated the TRR of AMSTAR to evaluate the importance of its measurement and contribute to the discussion of the measurement properties of AMSTAR and other quality assessment tools.

**Methods:**

Seven raters at three institutions independently assessed the methodological quality of SRs in the field of occupational health with AMSTAR. Between the first and second ratings was a timespan of approximately two years. Answers were dichotomized, and we calculated the TRR of all raters and AMSTAR items using Gwet’s AC1 coefficient. To investigate the impact of variation in the ratings over time, we obtained summary scores for each review.

**Results:**

AMSTAR item 4 (Was the status of publication used as an inclusion criterion?) provided the lowest median TRR of 0.53 (moderate agreement). Perfect agreement of all reviewers was detected for AMSTAR-item 1 with a Gwet’s AC1 of 1, which represented perfect agreement. The median TRR of the single raters varied between 0.69 (substantial agreement) and 0.89 (almost perfect agreement). Variation of two or more points in yes-scored AMSTAR items was observed in 65% (73/112) of all assessments.

**Conclusions:**

The high variation between the first and second AMSTAR ratings suggests that consideration of the TRR is important when evaluating the psychometric properties of AMSTAR.. However, more evidence is needed to investigate this neglected issue of measurement properties. Our results may initiate discussion of the importance of considering the TRR of assessment tools. A further examination of the TRR of AMSTAR, as well as other recently established rating tools such as AMSTAR 2 and ROBIS (Risk Of Bias In Systematic reviews), would be useful.

**Supplementary Information:**

The online version contains supplementary material available at 10.1186/s12874-021-01231-y.

## Background

Systematic reviews (SRs) can build the groundwork for evidence-based health care decision-making. They can provide the highest level of evidence, but they are not free from methodological flaws and biases. Consequently, biased SRs may lead to biased conclusions and might produce misleading prioritization in health care decision-making [1].

As the number of SRs is rapidly increasing [1, 2], overviews of multiple SRs of a related research question are conducted to overcome the problem of the increasing volume of SRs. Such overviews compile and provide a ‘user-friendly’ summary for decision-making [3]. To ensure an adequate quality of used and included SRs, instruments to assess the methodological quality and the risk of bias in SRs are crucial. They should provide valid results and good reliability measures.

TRR or intrarater reliability measures the agreement of ratings performed by one rater performing the same measurement with the same tool test object over a given timespan. In other words, it measures the rater self-consistency in scoring of a subject [4]. It allows us to draw conclusions about the reproducibility of a measurement tool.

AMSTAR (A Measurement Tool to Assess Systematic Reviews) is a widely used tool that was developed to assess the methodological quality of SRs [5] and is also commonly used to assess the quality of the included studies in overviews [6]. It consists of eleven items to assess the methodological rigor at different stages of the SR developmental process (see Additional File 1). More recently, a revised version of AMSTAR, called AMSTAR 2, was published in 2017 [7]. It was developed in response to studies discussing critical points and constraints of AMSTAR [8–10]. AMSTAR’s psychometric measurement properties, including reliability, validity and feasibility, were evaluated in many studies and in an SR [11]. However, data on reliability in terms of the test-retest reliability (TRR) of AMSTAR are still lacking.

To the best of our knowledge, no studies have investigated the TRR of AMSTAR and of quality assessment instruments for SRs in general. We considered investigating the TRR of AMSTAR because it is a well-established instrument, and to date, has been the most frequently used tool for quality assessment of SRs. Additionally, all reviewers involved in our study had some experience in using AMSTAR. With our study, we hope to contribute to the discussion of the measurement properties of AMSTAR and other quality assessment tools.

## Methods

This manuscript is part of a larger research project. The original (unpublished) study (hereafter termed “index study”) investigated the interrater-reliability of AMSTAR and R (evised)-AMSTAR) in SRs in the field of occupational health [12]. Two studies already used the resulting study pool of this study [13, 14]. The number of researchers involved in these studies varied between 5 and 7 depending on their availability. The present study is also based on the study pool of the index study. For further information on the index study we refer to one of the former publications [13, 14]. There was no protocol published a priori.

### Study selection

A systematic search was performed in the bibliographic database MEDLINE (via PubMed) and the Cochrane Database of Systematic Reviews (via the Cochrane Library) until 12.2014.

We included SRs in the field of occupational health that were published between 2010 and 2014, and included at least one randomized controlled trial.

### Quality assessment

Seven raters at three institutions independently assessed the methodological quality of the SRs with AMSTAR in an a priori-determined order. The first rating was performed in 2015/2016. No calibration exercise was performed in advance. Only for AMSTAR-item 1 was there an a priori agreement that the item should be rated “yes” if a study protocol existed. In 2018, after approximately two years (depending on when the assessment was completed), the same reviewers again rated the SRs with AMSTAR independently in the same order. The conduction of the present study was explained to all researchers in advance, namely rating the same SRs once again independently from the first rating.

### Reviewer experience

To evaluate whether the reviewers’ experience had an influence on TRR, we asked the reviewers to provide a self-assessment of their experiences before the first AMSTAR assessment started. The questionnaire included questions regarding their work experience in the field of evidence-based healthcare (in years), the number of SRs assessed with either AMSTAR, revised AMSTAR (R-AMSTAR) [15] or the Overview Quality Assessment Questionnaire (OQAQ) [16], and the number of SRs assessed with any other instruments (e.g., the SIGN (Scottish Intercollegiate Guidelines Network) checklist).

### Data analysis

The ratings of the 11 AMSTAR items were dichotomized into yes versus all other answers. We used descriptive statistics to describe the AMSTAR ratings. To overcome kappa paradoxes, we calculated the TRR of all raters using Gwet’s AC1 coefficient [17]. Finally, we interpreted the strength of agreement according to Landis and Koch as follows: poor (< 0), slight (0–0.2), fair (0.21–0.40), moderate (0.41–0.60), substantial (0.61–0.80) and almost perfect (0.81–1.00) [18].

### Summary scores of AMSTAR

To descriptively illustrate the impact of variation in AMSTAR ratings over time, we used the summary scores proposed by Banzi et al. [19]. Therefore, we compared the number of “yes-scored” items of the first rating with that of the second rating after two years. According to this classification scheme, a summary score between 8 and 11 indicates a high-quality SR. A moderate quality is assigned to a score of 4–7 and an SR of low quality to a score between 0 and 3 [19].

### Software

R statistical software was used to calculate Gwet’s AC1 [4].

## Results

We included 16 studies published between 2010 and 2014. The number of included RCTs varied between 3 and 57 with a median of 9. A meta-analysis was performed in ten reviews. The numbers of Cochrane and non-Cochrane reviews were intentionally chosen at a 1:1 ratio because the original data for our study form part of a larger project. The characteristics of the included SRs can be found in Additional file 2.

### Level of experience of the raters

All reviewers involved in our study have several years of working experience in the field of evidence-based health care (median: 7 years, range: 4–13 years, IQR: 5–7). Their work included conducting SRs and assessing the quality of SRs. At baseline the median number of SRs assessed with AMSTAR, R-AMSTAR or OQAQ was 15 (range: 1–80 assessments, IQR: 10–20), and the median number of SRs assessed with any other tool was 15 (range: 5–50 assessments, IQR: 10–25) (see Additional file 4).

### Results of TRR

The median TRR per AMSTAR item ranged between 0.53 and 1. Perfect agreement of all reviewers was observed for AMSTAR-item 1 with a Gwet’s AC1 of 1, which represented perfect agreement. The lowest median TRR of 0.53 (moderate agreement) was observed for AMSTAR item 4 (Was the status of publication used as an inclusion criterion?). Items 5 (Was a list of studies (included and excluded) provided?) and 10 (Was the likelihood of publication bias assessed?) provided a TRR of 0.6, also corresponding to moderate agreement [18].

The median TRR of the single raters showed high variability (range: 0.69–0.89). According to the classification of Landis and Koch, these values equated to a substantial to almost perfect level of agreement. The lowest TRR of all single ratings was − 0.02, and the highest was 1. Negative values of reliability measures indicate that coders are doing worse than coin flipping, indicating that at least some structural error exists. Mostly, it is due to structural misunderstanding between reviewers, indicating that there is a strong need for clarification [20]. The TRR per item and per rater is shown in Table 1.

**Table 1:** Median Test-retest-reliability (Gwet’s AC1) per Amstar item 1–11 reviewer (R1-R7), for *n* = 16 SRs

AMSTAR-Item	1	2	3	4	5	6	7	8	9	10	11	Median (Range)
**R1**	1	0.72	0.64	0.75	0.9	1	0.86	0.75	0.64	0.63	0.92	0.75 (0.63–1)
**R2**	1	0.9	0.92	0.63	0.89	0.75	1	0.68	0.84	0.64	1	0.89 (0.63–1)
**R3**	1	0.8	0.84	0.34	0.63	0.34	0.86	0.75	0.5	−0.02	0.93	0.75 (0.2–1)
**R4**	1	0.63	0.82	0.63	0.88	1	0.86	0.4	0.82	0.88	0.72	0.82 (0.4–1)
**R5**	1	0.8	0.8	0.6	0.53	0.86	0.58	0.69	0.82	0.5	−0.02	0.69 (−0.02–1)
**R6**	1	0.75	0.77	0.53	0.15	0.4	0.93	0.68	1	0.76	0.72	0.75 (0.4–1)
**R7**	1	0.88	0.92	0.88	0.51	0.77	1	0.68	0.53	0.6	0.75	0.77 (0.51–1)
**Median (Range)**	1 (1–1)	0.8 (0.63–1)	0.82 (0.64–1)	0.6 (0.53–1)	0.53 (0.15–1)	0.77 (0.34–1)	0.86 (0.58–1)	0.68 (0.4–1)	0.82 (0.5–1)	0.6 (0.2–1)	0.72 (−0.02–1)	

Legend: light gray: moderate agreement, medium dark colored: substantial agreement, dark colored almost perfect agreement

There was no association between TRR and years of working experience of the raters. Researchers who indicated a higher number of SRs assessed with AMSTAR, R-AMSTAR or OQAQ did not yield a higher median TRR and vice versa (see Additional file 4).

### Differences in summary scores

In 35% (39/112) of the assessments of the reviews, there was no difference in the summary scores over time. In 65% (73/112) of the assessments, the AMSTAR rating was different after two years. A change quantified by one point occurred in 38 assessments, whereas a change of two points occurred in 27. In eight cases, the judgment was changed by three or more points. In Table 2, the changes were quantified, and the direction (up- or downgrade) is presented.

**Table 2:** Changes in summary scores between first and second rating with AMSTAR for n = 16 SRs

	R1	R2	R3	R4	R5	R6	R7	Total
**no change ↔**	6	9	5	6	2	5	5	39
**1 point ↑**	4	2	6	1	1	4	4	22
**1 point ↓**	2	1		4	4	3	2	16
**2 points ↑**	1		3	2	2	1	3	12
**2 points ↓**	2	3		3	3	3	2	16
**3 points ↑**			2					2
**3 points ↓**	1	1			2			4
**4 points ↑**								
**4 points ↓**					4			4
**5 points ↑**								
**5 points ↓**					1			1
**Change in quality ↑**		1	5	2	3	1	2	14
**Change in quality ↓**	2	3		3	5	1	1	15

Legend: Changes from the first rating (2015/16) to second rating (2018) and resulting changes in overall quality classification

Regarding the quality classification based on the AMSTAR summary scores in 29 assessments, the differences in the scores revealed different judgment of the overall quality of the SR. Downgrading of the quality occurred in 15 cases, whereas in 14 assessments, the quality was upgraded (see Table 2). Most often, a change in the overall quality assessment was noticed in non-Cochrane reviews (*n* = 22 versus *n* = 7) (see Additional file 3).

## Discussion

### Main findings

Great variation was observed in the single TRRs of AMSTAR items as well as among the reviewers. The median TRR of the AMSTAR items ranged between 0.53 (moderate) and 1 (perfect agreement). The median TRR of the single raters ranged between 0.69 (substantial agreement) and 0.89 (almost perfect agreement). The rater with the highest variability between the first and second ratings provided TRRs between − 0.02 and 1 (see Table 1).

The most important aspect of our study could be seen when we focus on the great variation in the summary scores of the SRs. Variation of two or more points of yes-scored AMSTAR items was observed in 31% (35/112) of all assessments. However, AMSTAR is not intended to generate an overall score, and the meaningfulness of the overall score is questionable. However, especially when using AMSTAR summary scores as an indication for quality judgments and as an inclusion criterion in health care decision-making, e.g., for guidelines or for overviews, bias may be introduced when great variation in TRR is provided [21]. In a study about the impact of different inclusion criteria for overviews, the authors defined one criterion (the highest-quality SR) as the SR with the highest AMSTAR score (x/11) [21]. The study concluded that different inclusion decisions affect the comprehensiveness and results of overviews.

Studies have emphasized the harmfulness of suboptimal SRs and meta-analyses given the major prestige and influence these types of studies have acquired [1]. In nearly one-third (29/112) of all assessments, the change in the AMSTAR summary score resulted in an up- or downgrade of the reviews´ quality category. This fact underlines the importance of reliable and valid measurement tools to investigate the methodological quality of SRs to build a sound evidence base to inform decision-making. Taking this into account, we should expect a measurement tool for assessing the methodological quality of SRs to provide a high degree of self-consistency of the reviewers’ evaluation and thus almost perfect agreement. One reason for our findings might be the length of the timespan, which was two years. The literature about a suitable timespan when measuring the TRR indicates that a timespan that is too short might lead to a memory effect. On the other hand, if the timespan is too long, the ratings might be affected by the learning curve of the reviewers as well by changes in the field, such as the development of new assessment instruments [22, 23]. Studies in other research fields used varying timespans when measuring TRR, such as two weeks, 18 months and two years [24, 25]. The optimal timespan depends on the context in which the testing takes place. The AMSTAR instrument as well as the SRs was stable and consistent over the timespan. None of the reviewers were absolutely new in using AMSTAR, so they could be called well trained in using the tool. Therefore, the learning curve might not have had a substantial impact, and we might have expected only a small degree of change in the AMSTAR ratings. However, most research on reliability has been done in the field of psychology and other fields, so the test and test objectives differ, and transferability to our study might be limited.To the best of our knowledge, there is no “official” threshold up to which degree a TRR is still acceptable. COSMIN (COnsensus-based Standards for the selection of health Measurement INstruments), which is a guideline for selecting outcome measurement instruments for outcomes included in a core outcome set (COS), considered a reliability with an ICC or weighted kappa of ≥0.7 as a criterion for a good measurement property, which equates to substantial agreement according to Landis and Koch. Another study also refers to this value [26]. However, these studies refer only to interrater reliability [27].

### Our findings in context

In our study, we did not detect an association between reviewer working experience and TRR. More reviewer experience in terms of working experience and number of assessed reviews did not reflect a higher TRR or fewer changes in overall summary scores. This was also reported in another study connecting reviewer experience with their IRR when performing AMSTAR quality assessments [13].

### Strengths and limitations

To the best of our knowledge, the aspects of the TRR of AMSTAR have never been evaluated before. With seven reviewers at three different institutions and with different years of working experience and numbers of assessed reviews, we provide a number of factors that might have an influence on the outcomes.

Our study has several limitations. First, the number of included SRs for quality assessment was low, and the results might have differed if a higher number of studies had been assessed. However, our sample of reviews depicted Cochrane and non-Cochrane reviews at a 1:1 ratio. We did not perform a sample size calculation but our sample size of 16 reviews does correspond to a 25% error regarding raw agreement [4]. A further limitation is that we do not know if a timespan of approximately two years between the first and second quality assessments might be adequate. A shorter timespan might have provided different results. Other studies investigating TRR used various ranges of timespans, such as weeks, months and even years [24, 25, 28, 29]. In our study, all reviewers were experienced in using AMSTAR, and the learning curve might explain only a small degree of the variation. Another limitation is that we did not assess factors that might have influenced the raters’ performance between the two ratings. The number of quality assessments performed, the introduction of new tools (e.g. AMSTAR 2) as well as education might have played a role. However, the development and publication of AMSTAR 2 in 2017, which is a further developed version of AMSTAR and retains ten of the original domains, might have had an influence on the second rating [7]. Another limitation may be that we obtained an overall summary score of AMSTAR, which is not recommended by the developers of AMSTAR [7]. However, we only used the summary score descriptively to illustrate the impact of differences between the first and second quality ratings leading to different quality classifications and consequently long-term perspectives for making a different decision in health care. The overall score should be seen as a very rough estimate, taking into account that it is based on the assumption that all “yes-scored” AMSTAR items are equally important. Nevertheless, with our study, we contribute to the discussion on the intrarater reliability of quality assessment tools and point out the relevance of assessing it. The TRR of quality assessment tools should play a greater role in the discussion of psychometric properties of other instruments, such as AMSTAR 2 and ROBIS. A further limitation might be that in our study, we did not perform a calibration exercise a priori. We only calibrated AMSTAR item one a priori, which always yielded perfect agreement. The performance of a calibration exercise might have had an influence on the outcomes of our study, as many studies have emphasized its usefulness [30]. In particular, the AMSTAR items that showed a moderate TRR in our study are items where a calibration exercise might have been useful. Low reliability is often due to problems in the understanding of an item and accompanying guidance on how to apply it in combination with a calibration phase before each rating would be helpful to overcome this problem [11]. However, we did not test the understanding of the quality assessment tool. Studies that investigated the IRR of AMSTAR 2 concluded that an a priori calibration exercise should be performed [30, 31]. When investigating TRR, a calibration exercise should have been performed before the first and second ratings to provide the most benefit. In general, how wide the scope of the interpretation of the items is should be discussed to ensure consistency. One more limitation is that we used the publication of Landis and Koch to classify the TRR. We used Gwet’s alpha coefficient to calculate TRR, and Gwet does not recommend using the Landis and Koch classification [4]. However, the classification according to Landis and Koch is commonly used and facilitates readers´ ability to understand our results. Another limitation might be, that our study is based only on SRs in the field of occupational health and the generalizability of our results might be limited.

### Conclusion

To date, test-retest reliability (intrarater reliability) has been a neglected issue when evaluating the measurement properties of quality assessment tools (i.e., AMSTAR). Our study focused on the evaluation of the TRR of AMSTAR. Our results show that consideration of the TRR is important and that more evidence is needed The moderate TRR of AMSTAR items in our study raises the question of whether the consistency is moderate because the tool itself provides an excessively large scope of interpretation of the single items. However, our study emphasizes the need for an a priori calibration exercise, especially if there are different teams of researchers at different institutions, but for teams that have some shared working experience, a calibration exercise might overcome learning curve effects.

Finally, the importance of performing an a priori calibration exercise must be considered, which seems to have an important influence on the TRR as well as the IRR of AMSTAR.

Our study will contribute to the discussion of the importance of the TRR of assessment tools. A further examination of the TRR of AMSTAR 2 and ROBIS with a shorter timespan between the ratings would be useful.

## Supplementary Information

**Additional file 1.**

**Additional file 2.**

**Additional file 3.**

**Additional file 4.**

## Data Availability

The datasets used and/or analysed during the current study are available from the corresponding author on reasonable request.

## Abbreviations

AMSTAR: A Measurement Tool to Assess Systematic Reviews
IRR: Interrater reliability
OQAQ: Overview Quality Assessment Questionnaire
R-AMSTAR: revised AMSTAR
RCTs: randomized controlled trials
ROBIS: Risk Of Bias In Systematic reviews
SIGN: Scottish Intercollegiate Guidelines Network
SRs: Systematic Reviews
TRR: Test retest reliability
