# Supplementary material for: Measuring test-retest reliability (TRR) of AMSTAR provides moderate to perfect agreement – a contribution to the discussion of the importance of TRR in relation to the psychometric properties of assessment tools
Source: BMC Med Res Methodol. 2021 Mar 11;21:51. doi: 10.1186/s12874-021-01231-y (PMC7953720; doi:10.1186/s12874-021-01231-y)
Supplement: Supplementary file 1 — Additional file 1. [file 12874_2021_1231_MOESM1_ESM.docx]

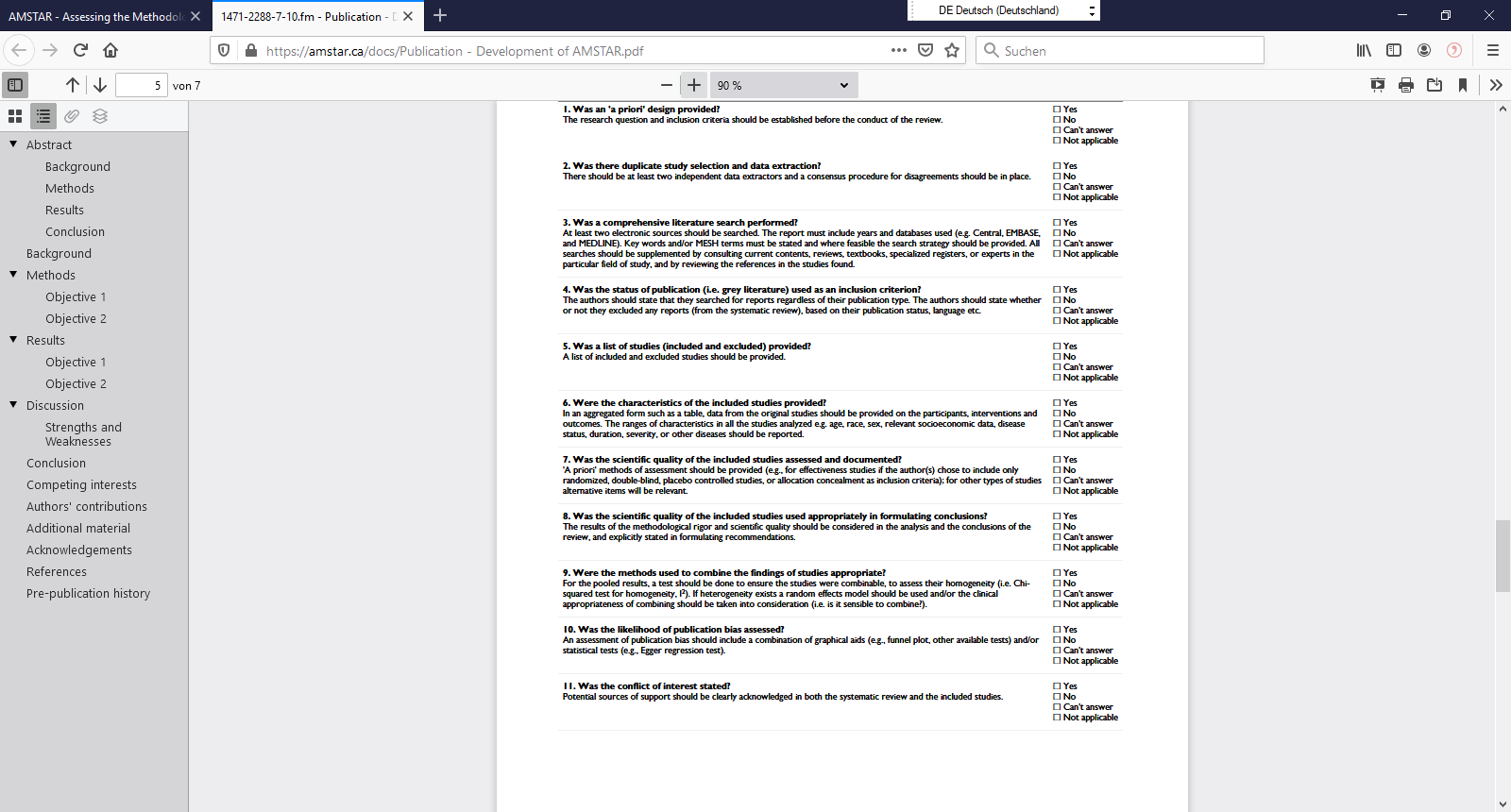
Additional File 1: AMSTAR checklist (1)

1. Shea BJ, Grimshaw JM, Wells GA, Boers M, Andersson N, Hamel C, et al. Development of AMSTAR: a measurement tool to assess the methodological quality of systematic reviews. BMC Med Res Methodol. 2007;7:10.
