# Supplementary material for: Measuring test-retest reliability (TRR) of AMSTAR provides moderate to perfect agreement – a contribution to the discussion of the importance of TRR in relation to the psychometric properties of assessment tools
Source: BMC Med Res Methodol. 2021 Mar 11;21:51. doi: 10.1186/s12874-021-01231-y (PMC7953720; doi:10.1186/s12874-021-01231-y)
Supplement: Supplementary file 3 — Additional file 3. [file 12874_2021_1231_MOESM3_ESM.docx]

Additional file 3: Differences between first and second rating which lead to a different overall score of the review

Legend: Dark grey = Reviews with differences;
First column: Middle grey = Cochrane Reviews; Light grey: Non-Cochrane Reviews
