# Supplementary material for: Measuring test-retest reliability (TRR) of AMSTAR provides moderate to perfect agreement – a contribution to the discussion of the importance of TRR in relation to the psychometric properties of assessment tools
Source: BMC Med Res Methodol. 2021 Mar 11;21:51. doi: 10.1186/s12874-021-01231-y (PMC7953720; doi:10.1186/s12874-021-01231-y)
Supplement: Supplementary file 4 — Additional file 4. [file 12874_2021_1231_MOESM4_ESM.docx]

Additional file 4: Experience of reviewers at the first rating and TRR

| Rater | Working experience in the field of evidence- based healthcare (in years) | Number of SRs assessed with either AMSTAR, R-AMSTAR or OQAQ | Number of SRs assessed with any other tool | TTR |
| --- | --- | --- | --- | --- |
| R1 | 4 | 20 | 15 | 0.75 |
| R2 | 4 | 20 | 15 | 0.89 |
| R3 | 6 | **80** | 15 | 0.75 |
| R4 | 7 | 10 | 5 | 0.82 |
| R5 | **13** | 15 | 35 | 0.69 |
| R6 | 7 | 10 | **50** | 0.75 |
| R7 | 7 | 1 | 5 | 0.77 |
| Median | 7 | 15 | 15 |  |

Legend: bold = highest experience
